# Comparison of electrohysterogram signal measured by surface electrodes with different designs: A computational study with dipole band and abdomen models

**DOI:** 10.1038/s41598-017-17109-3

**Published:** 2017-12-11

**Authors:** Pei Gao, Dongmei Hao, Yang An, Ying Wang, Qian Qiu, Lin Yang, Yimin Yang, Song Zhang, Xuwen Li, Dingchang Zheng

**Affiliations:** 1 College of Life Science and Bioengineering, Beijing University of Technology, Beijing, 100124 China; 2 Health and Wellbeing Academy, Faculty of Medical Science, Anglia Ruskin University, Chelmsford, CM1 1SQ UK

## Abstract

Non-invasive measurement of uterine activity using electrohysterogram (EHG) surface electrodes has been attempted to monitor uterine contraction. This study aimed to computationally compare the performance of acquiring EHG signals using monopolar electrode and three types of Laplacian concentric ring electrodes (bipolar, quasi-bipolar and tri-polar). With the implementation of dipole band model and abdomen model, the performances of four electrodes in terms of the local sensitivity were quantified by potential attenuation. Furthermore, the effects of fat and muscle thickness on potential attenuation were evaluated using the bipolar and tri-polar electrodes with different radius. The results showed that all the four types of electrodes detected the simulated EHG signals with consistency. That the bipolar and tri-polar electrodes had greater attenuations than the others, and the shorter distance between the origin and location of dipole band at 20 dB attenuation, indicating that they had relatively better local sensitivity. In addition, ANOVA analysis showed that, for all the electrodes with different outer ring radius, the effects of fat and muscle on potential attenuation were significant (all p < 0.01). It is therefore concluded that the bipolar and tri-polar electrodes had higher local sensitivity than the others, indicating that they can be applied to detect EHG effectively.

## Introduction

Electrohysterogram (EHG) signals^1^ recorded non-invasively using electrodes attached on the abdominal surface of pregnant woman is used to represent the electrical activity triggering the mechanical contraction of the myometrium, which has been used to monitor the uterine contractions^2,3^. The latest studies have focused on the analysis of EHG signal propagation, including velocity, directionality^4–6^ and synchronization^7^. To better understand the mechanisms of signal propagation during uterine contraction, a multi-lead EHG recording in a 4-by-4 configuration has been performed by placing an array of Ag/AgCl monopolar cutaneous electrodes at abdominal surface^8^, with the ground and reference electrodes attached on each side of the iliac crests. A grid of 8-by-8 high-density monopolar electrodes on the midline of the lower abdomen immediately below the umbilicus has also been proposed, with the common reference placed on the right hip, close to the ground electrode^9^. The monopolar recordings have certain disadvantages, which capture interference from other physiological signals, including the abdominal muscle electrical activity, the electrocardiogram, respiratory movements, electrode-skin contact potential fluctuation, and movement artefacts from both mother and fetus. Due to the blurring effect of different conductivities of the volume conductor, the monopolar recordings have also been shown to have low spatial resolution for localizing and differentiating multiple dipole sources^9^, limiting their application in signal propagation studies^6,10^.

Laplacian potential recordings of EHG signals has thus been proposed to improve the spatial resolution of surface bioelectric signal recordings^11^. The Laplacian is the second spatial derivative of the potential recorded on the body surface, which can reduce the smoothing effect of the volume conductor and enhance the electrical activity at the detection location^11,12^. With the application of discretization techniques, such as the finite difference numerical approximation or spline Laplacian estimation algorithm, the Laplacian of a bioelectric potential can be estimated from the measurements by the monopolar electrodes on the body surface^13,14^ or by specially designed concentric ring electrodes. Three types of concentric ring electrodes (bipolar, quasi-bipolar and tri-polar) have been used to estimate the Laplacian of different bioelectric signal potentials, including the electrocardiogram (ECG)^15,16^, electroencephalogram (EEG)^12,13^ and the intestinal electrical activity^17^. Since EHG signals have similar characteristics as the above physiological signals, the use of Laplacian electrodes could also improve the quality of EHG recordings in comparison with the monopolar recordings. However, the performances of using Laplacian electrodes (including the Laplacian concentric electrodes of bipolar, quasi-bipolar and tri-polar) to record EHG signals have not been quantitatively assessed and compared with the monopolar electrode.

It is known that the contraction and relaxation of the uterine muscle (myometrium) result from the depolarization and repolarization of the muscle-cell membranes^9^, and the spontaneous electrical activity of the myometrium, which initiates from myometrium cells (pacemaker) and then excites to surrounding regions, can be measured on the abdominal surface as EHG^18–20^. To describe the propagation of excitability on myometrium, a dipole band model has been proposed to represent myometrium cells, from which the characteristics of electrical activity can be described^21,22^. To the best of our knowledge, the dipole band model has not been applied in a computational study to investigate the propagation of uterine activities.

Besides, the biological tissue between the electrical source at the myometrium and the recording site on the skin is regarded as a volume conductor^9^. It generates a low-pass filtering effect, which affects the spatial selectivity^23^. Therefore, the influence of biological tissue on the conduction of EHG signals to the abdominal surface of a pregnant woman can not be neglected.

The aim of this study is to evaluate the performance of recording EHG signals in terms of local sensitivity from four types of electrodes (the monopolar and the bipolar, quasi-bipolar and tri-polar Laplacian concentric electrodes) based on a computational abdomen model and a dipole band model of uterine activity, and assess the effects of fat and muscle thickness on the recorded EHG signals.

## Results

### Simulated EHG signal source with moving dipole band

**Figure 1:**
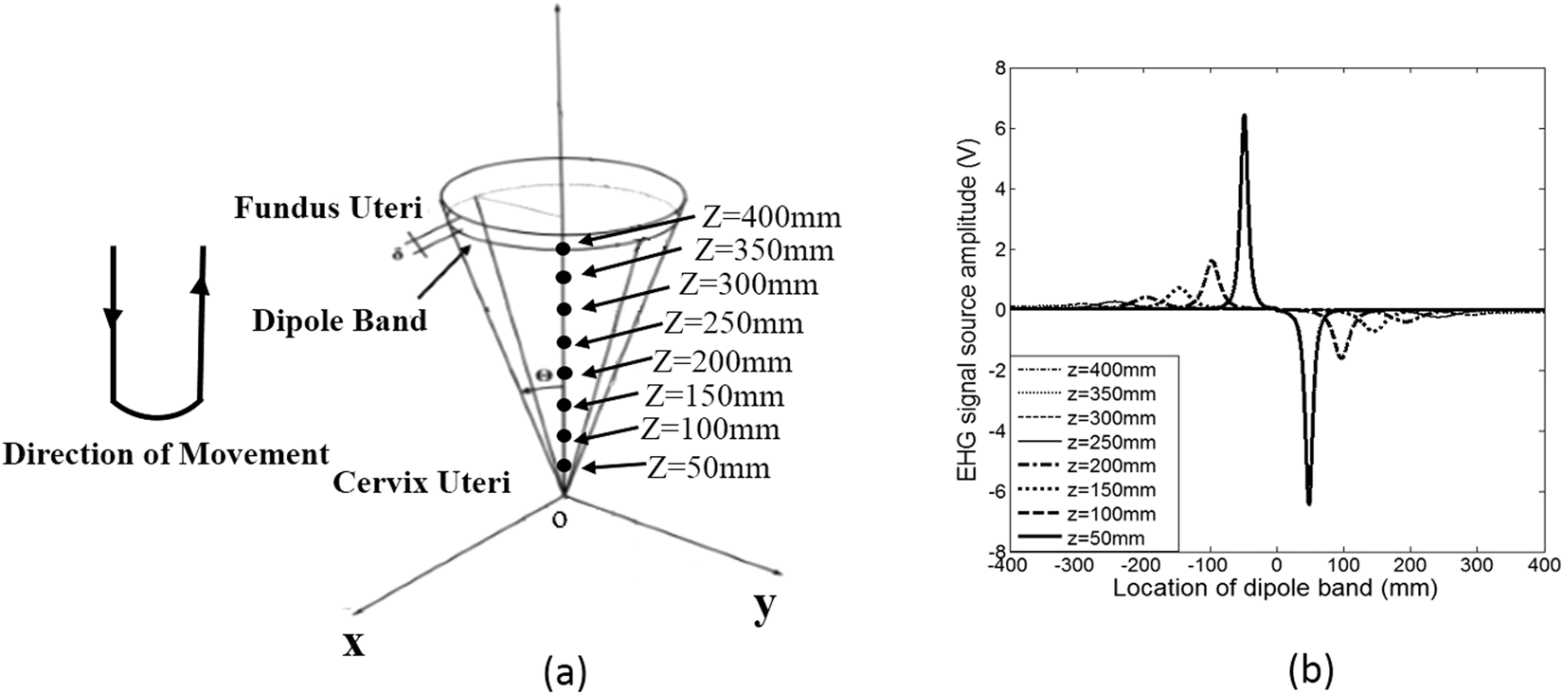
Simulated EHG signal sources with the dipole band moving from the fundus to cervix of the uterus, and returning to fundus. (**a**) Movement direction of the dipole band and the simulation position on the z axis. (**b**) The simulated EHG signal sources at different positions on z axis with moving dipole band. The horizontal axis from left to right (−400 mm to 400 mm) represents that the dipole band moves from the fundus to cervix of the uterus, and return to the fundus.

Figure 1 shows the simulated EHG signal sources at different positions on z axis, with the dipole band moving from the fundus to cervix of the uterus, and returning to the fundus. The moving dipole band started at −400 mm (moving toward cervix of the uterus) at time = 0s, and the propagation speed was set to 30 mm/s in the simulation, from formula (1) the movement time from the fundus to cervix was calculated to be 27 s.

### Comparison of EHG signal amplitude recorded by each of the four electrodes

**Figure 2:**
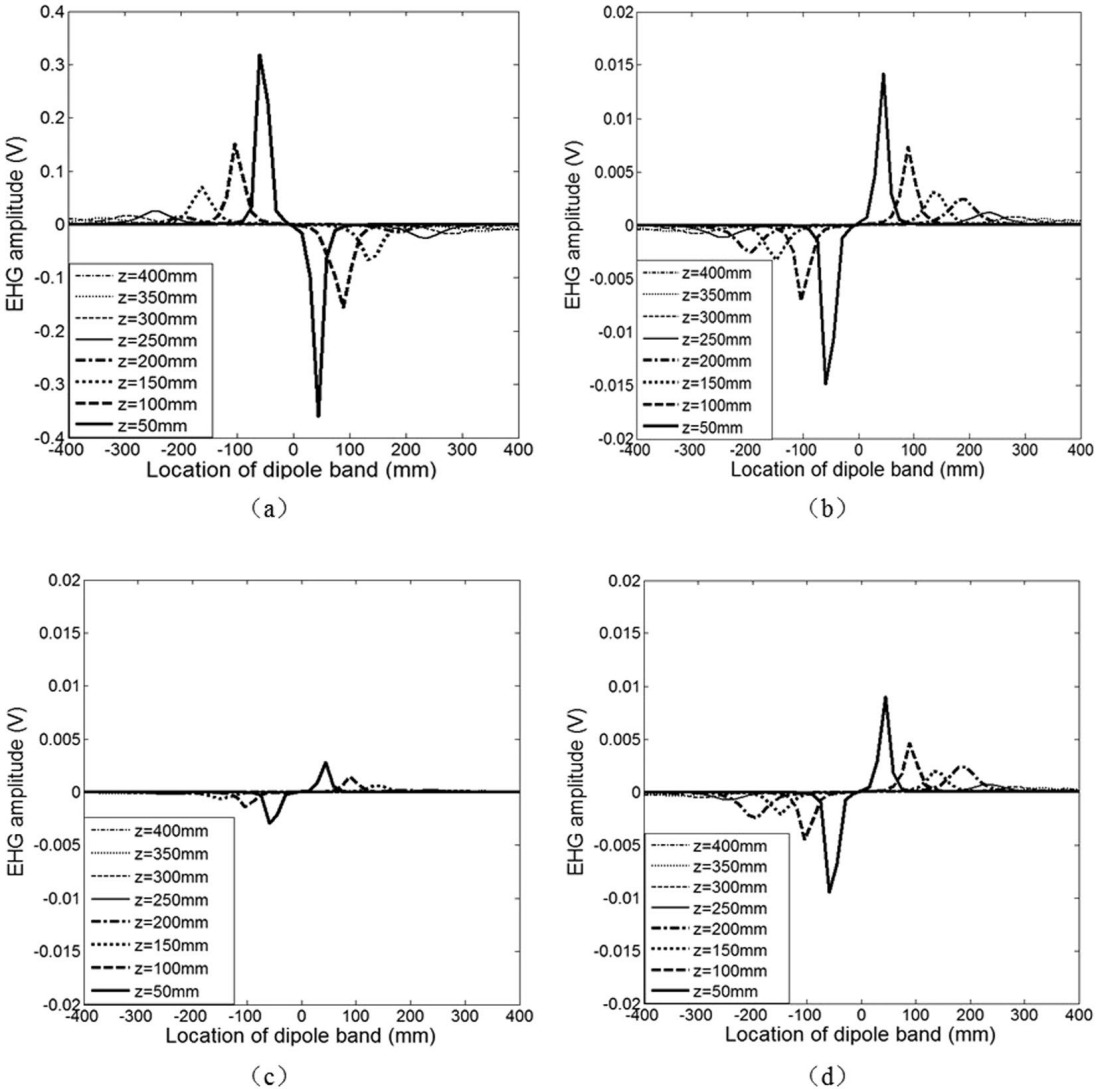
EHG signals recorded by the (**a**) monopolar, (**b**) bipolar, (**c**) quasi-bipolar, (**d**) tri-polar with the different positions and dipole band movement, as shown in the Fig. 1(a).

The EHG signals recorded by each of the four electrodes with the movement of dipole band are shown in Fig. 2. Because there was 1.78 s delay of EHG signals between adjacent positions of 50 mm, the propagation speed was therefore estimated to be 28 mm/s. Figure 2 also shows that the closer the dipole band to the origin or the coordinate center, the bigger peak of EHG signal amplitude. In addition, it can be seen that, the EHG peak amplitudes detected by the monopolar electrode were larger than the Laplacian electrodes, and all the peak amplitudes of EHG signals recorded by the four electrodes appeared at the same location of dipole band, suggesting the consistency of the changes of EHG amplitude between the four electrodes.

### Comparison of Laplacian potential attenuation with a moving dipole band

Three different outer ring radius of 10/15/20 mm were simulated for the analysis of local sensitivity of electrodes with a moving dipole band. Due to its symmetry, only the results with the dipole band moving from 0 to 30 mm along z axis was presented here.

**Figure 3:**
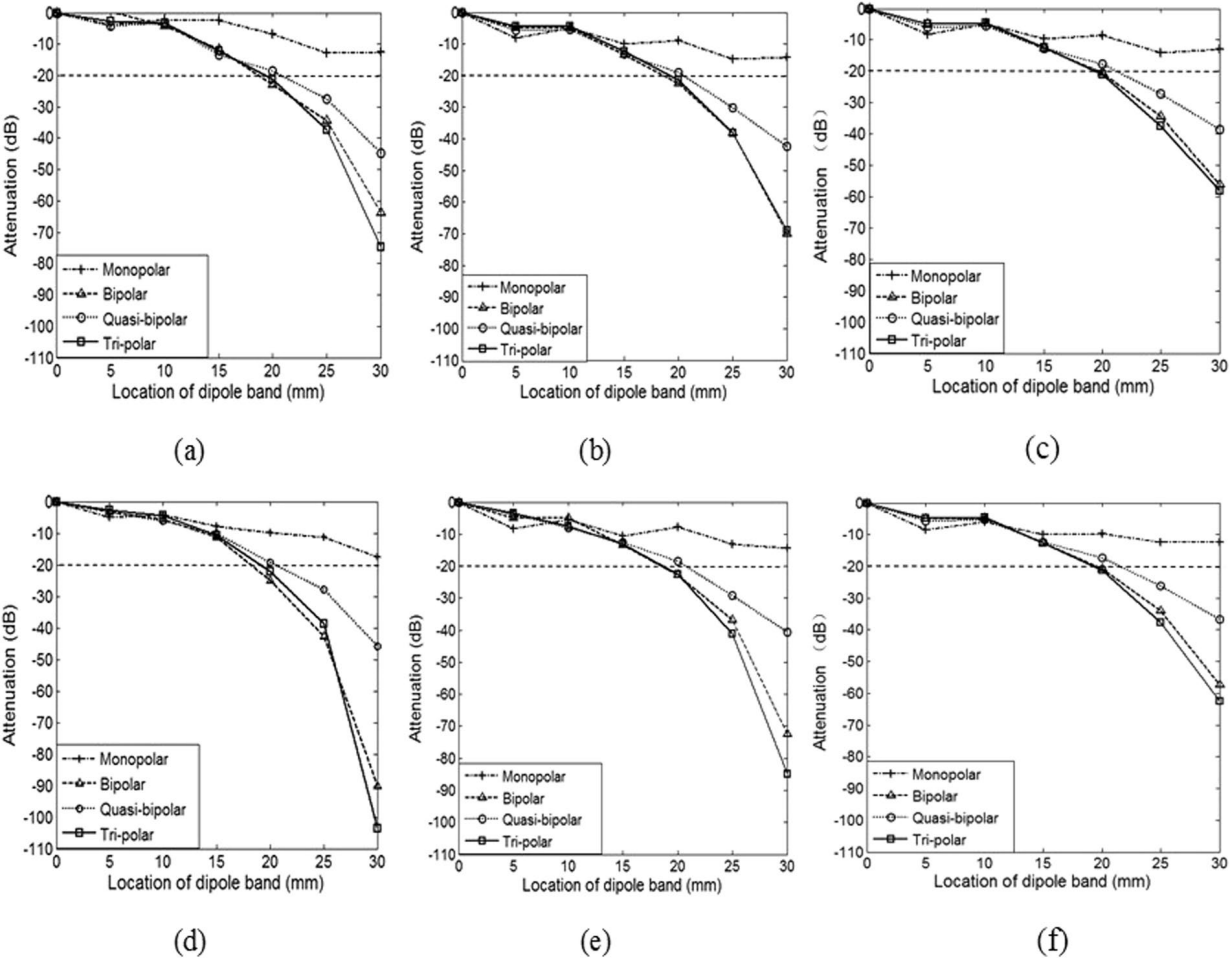
Comparison of Laplacian potential attenuations between the four types of electrodes without noise dipole (**a**,**b**,**c**) and with 6 noise dipoles (**d**,**e**,**f**). The performance with different outer ring radius was given separately. (**a**,**d**) outer ring radius 10 mm; (**b**,**e**) outer ring radius 15 mm; and (**c**,**f**) outer ring radius 20 mm. It can be seen that bipolar and tri-polar electrodes had larger attenuations than the other electrodes regardless of the outer ring radius.

Figure 3 shows Laplacian potential attenuations, separately for all the four electrodes without noise dipole (a,b,c) and with 6 noise dipoles (d,e,f), as well as for different outer ring radius. For all the four type of electrodes, the potential attenuation increased gradually with the dipole band moving away from the origin. The bigger outer ring radius resulted in smaller potential attenuation. The bipolar and tri-polar electrodes had shorter distance at 20 dB than the others, suggesting that the bipolar and tri-polar electrodes have higher local sensitivity, which were more sensitive to the local signal source than the monopolar and quasi-bipolar electrodes.

Therefore, according to the overall assessment of the local sensitivity, the bipolar and tri-polar electrodes were considered as the optimal electrodes for EHG recording, which were used for the further investigation.

### Effect of fat and muscle thickness on attenuation

**Figure 4:**
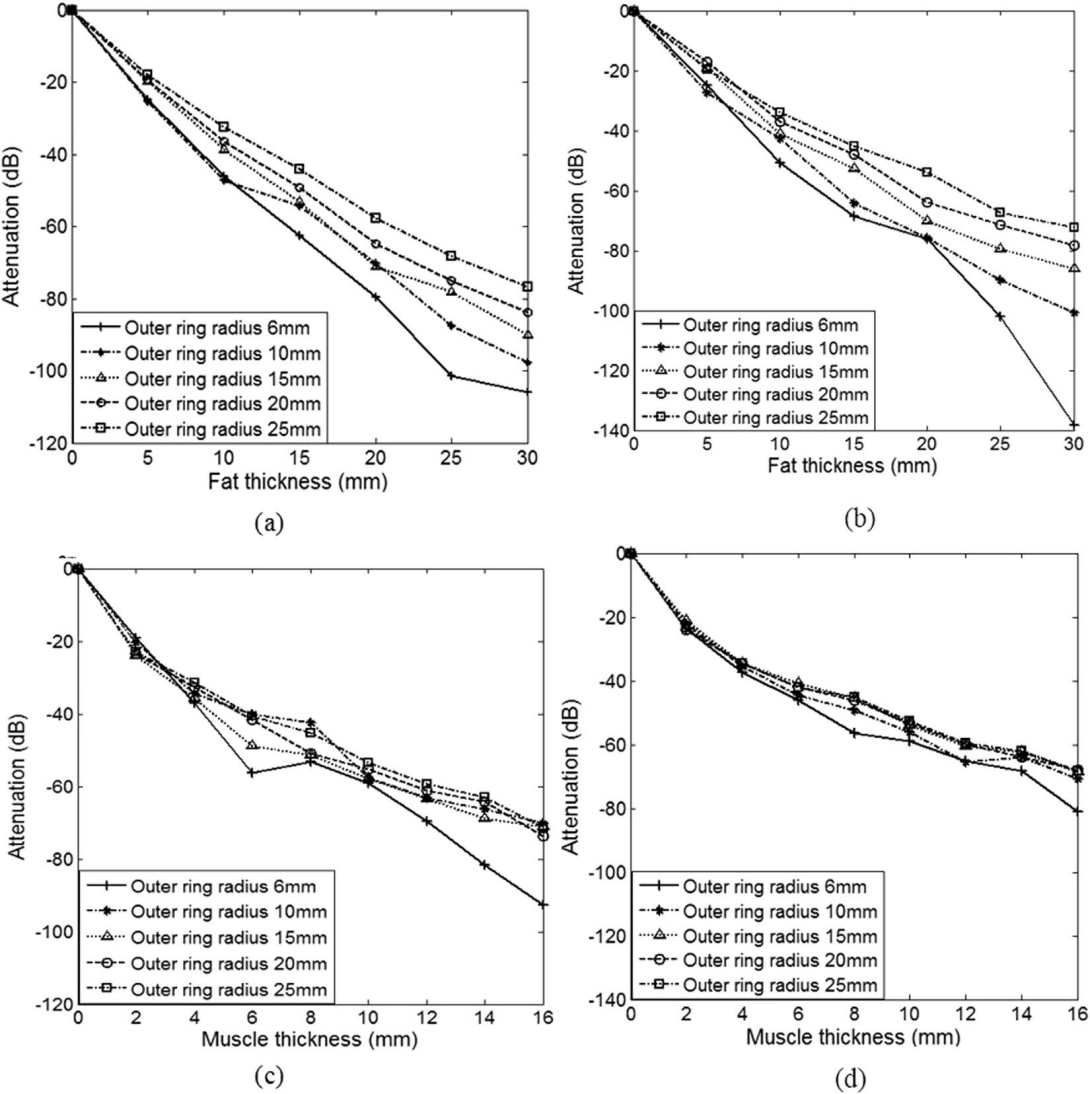
Attenuation changes with different fat thickness 0–30 mm and a fixed muscle thickness of 8 mm, (**a**) without noise, (**b**) with 6 noise dipoles; with different muscle thickness 0–16 mm and a fixed fat thickness of 15 mm, (**c**) without noise, (**d**) with 6 noise dipoles.

Statistical analysis showed that there was no significant difference between the bipolar and tri-polar electrodes on the attenuation with different fat and muscle thickness (all p > 0.05). For simplicity, only the results from the tri-polar electrode were exhibited below in details. As shown in Fig. 4, across all the different outer ring radius, ANOVA analysis showed that there was significant effect of the fat and muscle on attenuation (both p < 0.01). In details, Fig. 4(a) shows that the attenuations increased significantly with the increase of fat thickness. The same results were found even with 6 noise dipoles added, as shown in Fig. 4(b). In addition, as shown in Fig. 4(c) and (d), the attenuations increased with the increase of muscle thickness with or without noise.

## Discussion

This study has quantitatively compared the performance of monopolar electrode and three Laplacian electrodes for measuring EHG signals using a computational approach. To the best of our knowledge, this is the computational study for this investigation using the combined dipole band and abdomen models. The EHG signal sources were generated with the dipole band moving from the fundus to cervix of the uterus, and returning to the fundus. The amplitudes of EHG signals were recorded by different types of four electrodes, with their potential attenuations compared with different outer ring radius and with a moving dipole band. Overall, the Laplacian recordings with bipolar and tri-polar design had been shown to provide higher local sensitivity than the monopolar surface EHG recordings. The change of attenuations with the increase of fat and muscle thickness have also been investigated.

EHG signal sources were simulated with the dipole band moving from the fundus to cervix of the uterine, and then returning. The peak amplitude of EHG signal appeared at same location of dipole band for all the detections, which suggests the consistency of recording EHG from the four electrodes. However, the phase of EHG peaks recorded by the Laplacian electrodes and monopolar electrode were opposite. It can be explained by the different detection principle of the four electrodes, in which the potentials of Laplacian electrodes are the second derivative of potential. Although the EHG signals detected by Laplacian electrodes are smaller than by monopolar electrode, the uterine activity detected at the local region was enhanced with Laplacian electrodes, which is more meaningful in the analysis of uterine activity signal^24^, especially its propagation characteristics. Besides, the propagation velocity of EHG signals was 28 mm/s according to the inter-electrodes distance and time delay, which was very close to the preset velocity of 30 mm/s^25^.

With the moving dipole band, the potentials detected by the four electrodes with different outer ring radius attenuated as the dipole band moved away from the origin, and the smaller the outer ring radius, the more sensitive the electrode is. With different outer ring radius, the Laplacian electrodes exhibited larger attenuations when compared with the monopolar regardless of the dipole band location. These results suggested that the Laplacian electrodes have higher local sensitivity and better spatial resolution when compared with monopolar electrode. Besides, the EHG signals recorded by the bipolar and tri-polar attenuated quickly with a shorter distance from the origin than the other electrodes. These findings partially agreed with the published study^26^, where the tri-polar electrode had been indicated to significantly improve local sensitivity. Nevertheless, our simulation study with the dipole band model and the abdomen model has demonstrated that the bipolar and tri-polar electrodes achieved better performance for EHG recordings, providing guides for future electrode design.

Additionally, the effects of fat and muscle thickness on the attenuation of the EHG signals recorded by the bipolar and tri-polar with different outer ring radius have been investigated. Overall, regardless of the noises, the attenuations increased with the increase of fat thickness and muscle thickness. These findings can be partly explained by the tissue impedance changes with the increase of  fat and muscle thickness. In addition, the Laplacian potential was inversely proportional to the distance between the electrode and dipole band. The smaller the distance, the greater the potential was. Moreover, it was observed that the potential attenuations caused by fat were larger than by muscle. One possible explanation is that fat has larger relative permittivity and smaller conductivity than muscle, suggesting that the effect of fat on EHG signals is greater than muscle.

As an initial research, the uterine activity and the interference signals were simplified and simulated by the movement of the dipole band model. We supposed that the myometrium tissue was isotropic and the EHG signal propagated either along the longitudinal axis or the circumferential of the uterus. Although, the current model is still far away from the ideal situation and the recording of other signals would improve the models. However, as the focus of this study is on the performance comparison between different electrodes, not on the development of realistic model, we believe it is acceptable to estimate the electrodes performance. Besides, the uterus usually tilts to the right or left, not exactly in the middle of the abdomen, and we will consider to reconstruct the 3-D uterus with MRI data to approximate the real uterus in the future studies^27^. Finally, the narrower the ring width is, the smaller the error between the estimated Laplacian and analytical Laplacian^28^. Therefore, it would be better to have the ring width as narrow as possible. In our study, the ring width of the Laplacian electrodes is the same, allowing proper performance comparison between electrodes.

In conclusion, this study has computationally demonstrated that, in comparison with the monopolar electrode, the Laplacian electrodes had better local sensitive for EHG recordings, and the bipolar and tri-polar achieved better performance, indicating that it can be applied to detect EHG signals more effectively.

## Methods

### Electrode design

**Figure 5:**
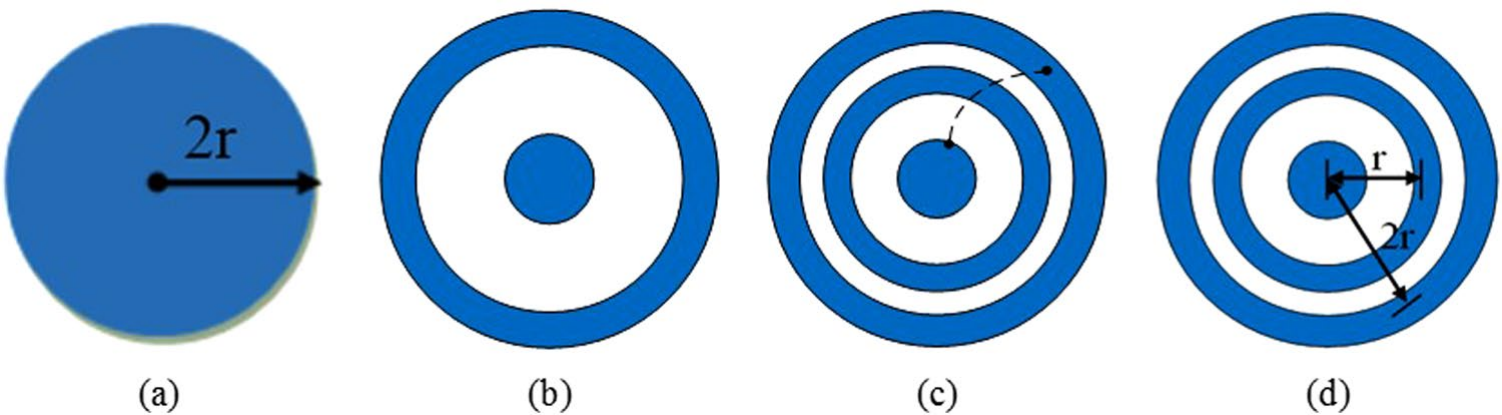
Four types of electrode designs: (**a**) Monopolar, (**b**) Bipolar, (**c**) Quasi-bipolar with shorted disc and outer ring, (**d**) Tri-polar concentric ring electrodes.

As shown in Fig. 5(a), the monopolar Ag/AgCl electrode with its radius of 2r = 6–26 mm was designed for the monopolar EHG recordings. The bipolar, quasi-bipolar and tri-polar concentric ring electrodes, as shown in Fig. 5(b)–(d) respectively, were also designed to acquire Laplacian potentials on the abdominal surface of a pregnant woman. The bipolar electrode consisted of a central disc and an outer ring. Both the quasi-bipolar and tri-polar electrodes had three elements including a central disc, a middle ring and an outer ring, while their difference was that the disc and outer ring were shorted for the quasi-bipolar. The inter-electrode distance between the central disc and the middle ring was r, which was doubled to the outer ring. Taking the actual uterus size into consideration, the outer ring radius was set to 2r = 6–26 mm, allowing at least two electrodes to be placed. The central disc radius was between 1.7–3.6 mm to ensure its impedance was equal to the middle rings, which would improve the common mode rejection ratio of the preamplifier^29^.

### Simulation of dipole band and abdomen models

#### Dipole band model

**Figure 6:**
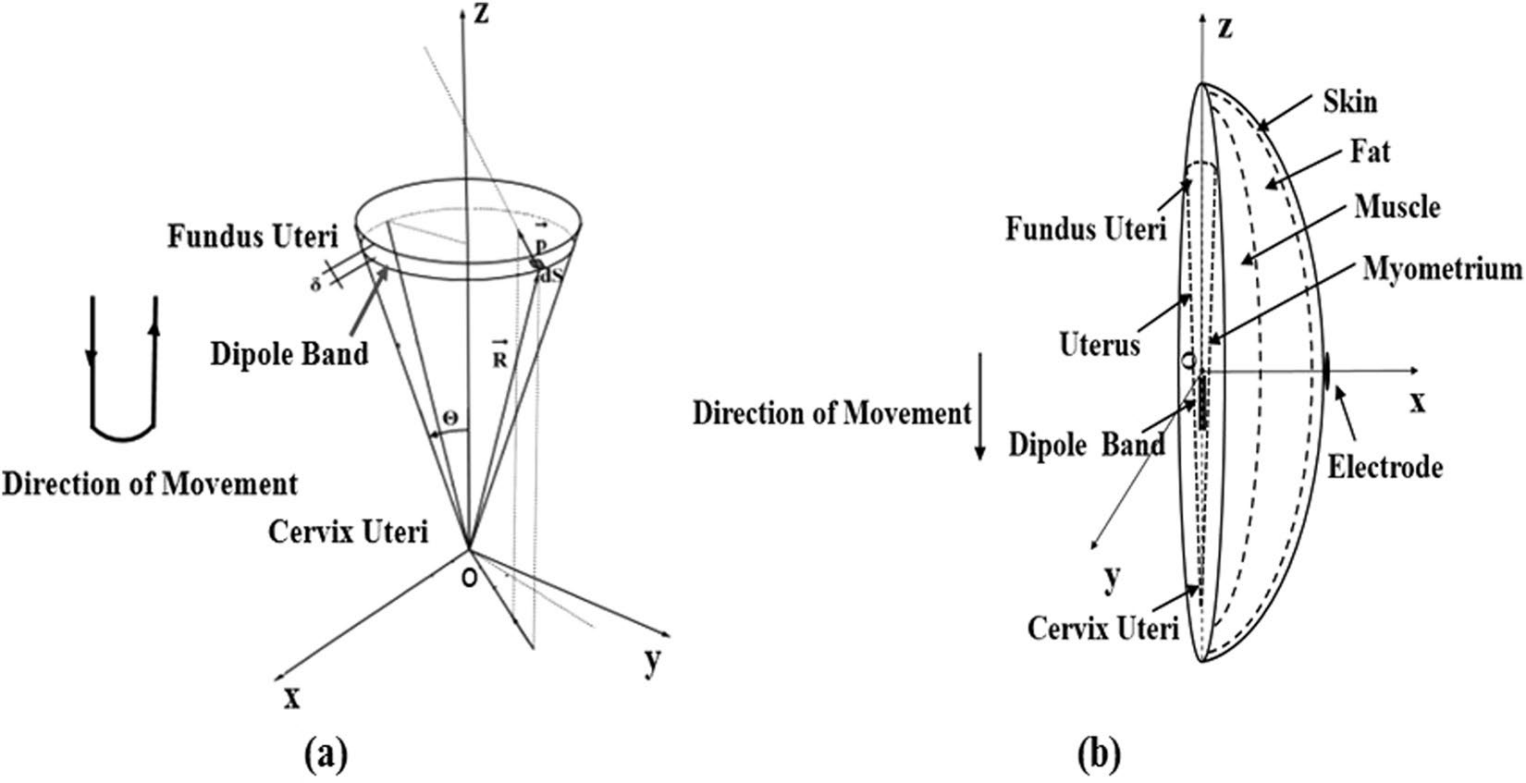
Illustration of (**a**) the modelled human uterus as an inverted cone with a dipole band moving from the fundus to cervix of the uterus and then returning to the fundus, and (**b**) the simulated abdomen model of a pregnant women, including human uterus and three tissue layers (skin, fat and muscle).

In order to reflect the electrical activity of myometrium cells^30^, the human uterus was modelled as an inverted cone with the dipole band moving from the fundus to cervix of the uterus and then returning to the fundus, as shown in Fig. 6(a). The simulated EHG signal source was obtained as follows^21,22,25^:

The displacement of dipole band is as:

1 $$z=c\,\ast \,t$$

where c is the velocity of dipole band of about 30 mm/s^19^ and t is the propagation time in second, t = 0, 1, 2, 3…

The position of the dipole band on the tapered wall is as:

2 $$\overrightarrow{R(z)}=\{\overrightarrow{Ros}-c\cdot t\}/\,\cos \,\theta $$

where $\overrightarrow{Ros}$ is the initial position of the dipole band on the z axis, it was set to 400 mm. According to the clinical data, on average, the uterus size (350 mm × 250 mm × 220 mm) of a pregnant women with a full-term pregnancy is about 5 times bigger than a non-pregnant woman, from which *θ* was estimated to 10° ^31^.

The area of the dipole band is as:

3 $$\overrightarrow{S(z)}=\pi \cdot \,\sin \,\theta \{{[\overrightarrow{R(z)}+\delta ]}^{2}-\overrightarrow{R{(z)}^{2}}\}$$

where $\overrightarrow{{p}_{0}}$ is the dipole band moment. Since the charge distribution on either side of the cell is about 1*10^−4^ C/mm^2^, $\overrightarrow{{p}_{0}}$ was set to 2.2 × 10^−13^ Cmm^21,22^. *δ* is the width of dipole band, and was set to 6 mm.

The density of dipole band is:

4 $$\overrightarrow{D(z)}=\frac{\overrightarrow{{P}_{0}}}{\pi \cdot \,\sin \,\theta \{{[\overrightarrow{R(z)}+\delta ]}^{2}-{\overrightarrow{R(z)}}^{2}\}}$$

The potentials of any point in z axis as:

5 $$v({\zeta }_{i})=-\frac{\overrightarrow{D(z)}}{2{\varepsilon }_{0}}\times \{\frac{{\zeta }_{i}-[R(z)+\delta ]\,\cos \,\theta }{{\{{{\zeta }_{i}}^{2}-2[R(z)+\delta ]{\zeta }_{i}\cos \theta +{[R(z)+\delta ]}^{2}\}}^{1/2}}-\frac{{\zeta }_{i}-R(z)\cos \,\theta }{{\{{{\zeta }_{i}}^{2}-2R(z){\zeta }_{i}\cos \theta +R{(z)}^{2}\}}^{1/2}}\}$$

where ${\zeta }_{i}$ is the position in z axis.${\varepsilon }_{0}$ is the conductivity of muscle, and was set to 0.36.

#### Abdomen model

To compare the performance of the four types of electrodes, the myometrium was set as the ground, and the center of uterus as the origin to establish rectangular coordinate system. The abdomen of a pregnant woman was modelled with a hemisphere with the radius of 100 mm, which had three-layer tissues (skin, fat and muscle). Besides, the abdomen model was placed in an infinite electrostatic field filled with air and the boundary potential was set to 0. The electrode was placed on the abdominal surface on the same horizontal level as the center of uterus, and the dipole band was placed close to the myometrium to simulate the uterine activity, which moved from the fundus to cervix of the uterus in z axis with 40 mm in length, see Fig. 6(b). Table 1 lists the abdomen model parameters for the simulation.

**Table 1:** Physical parameters of the abdomen tissues^9,35^.

Tissue	Thickness (mm)	Relative permittivity	Density (Kg/m3)	Atmospheric heat capacity (J/(Kg·K))	Heat conductivity coefficient (W/m·K)
Skin	2	40.0	1109	3391	0.37
Fat	15	12.7	911	2348	0.21
Muscle	8	66.2	1190	3421	0.49

#### Laplacian potential approximation

(a) Laplacian potential of bipolar concentric ring electrode.

**Figure 7:**
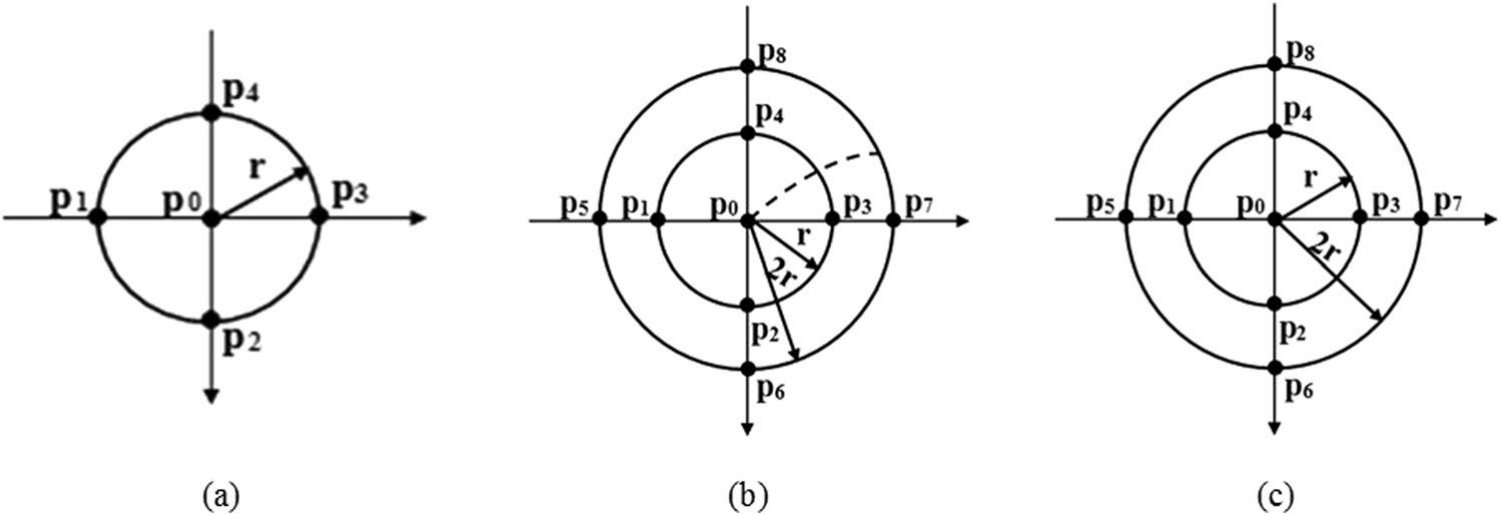
Different arrangements of the approximation of Laplacian potential of concentric ring electrodes. (**a**) five-point arrangement, (**b**) quasi-bipolar arrangement, (**c**) nine-point arrangement. p1, p2, p3, and p4 have the same spacing r to p0, and p5, p6, p7, and p8 have the same spacing 2r to p0. (**b**) Laplacian potential of quasi-bipolar concentric ring electrode.

A five-point arrangement was used to approximate the Laplacian potential detected by the bipolar concentric ring electrode. As shown in Fig. 7(a), it was formed by points p1, p2, p3, and p4 to p0 with the same spacing of r. The Laplacian potential v_i_ at point p_i_ was obtained using the Taylor series expansion along x/y axis and the finite difference approximation methods as formula (6)–(9)^13^

6 $${{\rm{v}}}_{{\rm{1}}}\cong {{\rm{v}}}_{{\rm{0}}}-r\frac{\partial v}{\partial x}{|}_{{\rm{p0}}}+\frac{1}{2}{r}^{2}\frac{{\partial }^{{\rm{2}}}{\rm{v}}}{\partial {{\rm{x}}}^{{\rm{2}}}}{|}_{{\rm{p0}}}$$

7 $${{\rm{v}}}_{{\rm{2}}}\cong {{\rm{v}}}_{{\rm{0}}}-{\rm{r}}\frac{\partial {\rm{v}}}{\partial {\rm{y}}}{|}_{{\rm{p0}}}+\frac{1}{2}{{\rm{r}}}^{2}\frac{{\partial }^{{\rm{2}}}{\rm{v}}}{\partial {{\rm{y}}}^{{\rm{2}}}}{|}_{{\rm{p0}}}$$

8 $${{\rm{v}}}_{3}\cong {{\rm{v}}}_{0}+{\rm{r}}\frac{{\rm{\partial }}{\rm{v}}}{{\rm{\partial }}{\rm{x}}}{|}_{{\rm{p}}0}+\frac{1}{2}{{\rm{r}}}^{2}\frac{{{\rm{\partial }}}^{2}{\rm{v}}}{{\rm{\partial }}{{\rm{x}}}^{2}}{|}_{{\rm{p}}0}$$

9 $${{\rm{v}}}_{4}\cong {{\rm{v}}}_{{\rm{0}}}+{\rm{r}}\frac{\partial {\rm{v}}}{\partial {\rm{y}}}{|}_{{\rm{p0}}}+\frac{1}{2}{{\rm{r}}}^{2}\frac{{\partial }^{{\rm{2}}}{\rm{v}}}{\partial {{\rm{y}}}^{{\rm{2}}}}{|}_{{\rm{p0}}}$$

where v_0_, v_1_, v_2_, v_3_, v_4_ are the potentials from point p_0_, p_1_, p_2_, p_3_ and p_4_. v_1_ and v_3_ are obtained along x axis, and v_2_ and v_4_ are obtained along y axis.

The sum of the four potentials is^32^:

10 $$\begin{array}{rcl}{{\rm{v}}}_{1}+{{\rm{v}}}_{2}+{{\rm{v}}}_{3}+{{\rm{v}}}_{4} & \cong  & 4{{\rm{v}}}_{0}+{{\rm{r}}}^{2}(\frac{{\partial }^{2}{\rm{v}}}{\partial {{\rm{x}}}^{2}}+\frac{{\partial }^{2}{\rm{v}}}{\partial {{\rm{y}}}^{2}}){|}_{P0}\\  & = & 4{{\rm{v}}}_{0}+{{\rm{r}}}^{2}{{\rm{\Delta }}v|}_{P0}\end{array}$$

Next, Δp_0_ was used to replace Δv|_p0_, the approximation to the Laplacian of potential at p_0_ was

11 $${{\rm{\Delta }}{\rm{p}}}_{{\rm{0}}}\cong \frac{4}{{{\rm{r}}}^{2}}(\overline{{\rm{v}}}-{{\rm{v}}}_{0})$$

where $\overline{{\rm{v}}}$ is the average of v_1_, v_2_, v_3_ and v_4_.

According to Husikamp^33^, the discrete equation (11) can be applied to the disc and concentric ring electrode system by performing the integral along the circle of radius r around the point p_0_ of the Taylor expansion and defining X = r cos(θ) and Y = r sin(θ)^33^, as shown in (12).

12 $${{\rm{\Delta }}{\rm{p}}}_{0}\cong \frac{4}{{{\rm{r}}}^{2}}(\frac{1}{2\pi }{\int }_{0}^{2{\rm{\pi }}}v({\rm{r}},\theta )d\theta -{{\rm{v}}}_{{\rm{0}}}=\frac{4}{{{\rm{r}}}^{2}}\overline{{{\rm{v}}}_{{\rm{r}}}}-{{\rm{v}}}_{0})$$

where $\overline{{{\rm{v}}}_{{\rm{r}}}}=\frac{1}{2\pi }{\int }_{0}^{2\pi }{\rm{v}}({\rm{r}},\theta ){\rm{d}}\theta$ is the average potential of the ring with spacing r.

Laplacian potential of the quasi-bipolar concentric ring electrode was estimated using quasi-bipolar method, as shown in Fig. 7(b). The potentials at p_5_ to p_8_ and p_0_ were averaged as they were shorted. Using the Taylor series expansion and the finite difference approximation methods, the Laplacian at p_0_ was given as:

13 $${{\rm{\Delta }}{\rm{p}}}_{0}\cong (\frac{{\partial }^{2}{\rm{v}}}{\partial {{\rm{x}}}^{2}}+\frac{{\partial }^{2}{\rm{v}}}{\partial {{\rm{y}}}^{2}}){|}_{P0}=\frac{4}{{{\rm{r}}}^{2}}[\frac{1}{2}(\frac{1}{4}\sum _{{\rm{i}}=5}^{8}{{\rm{v}}}_{{\rm{i}}}+{{\rm{v}}}_{0})-\frac{1}{{\rm{4}}}\sum _{{\rm{j}}=1}^{4}{{\rm{v}}}_{{\rm{j}}}]$$

This was generalized to quasi-bipolar concentric ring electrode as

14 $${{\rm{\Delta }}{\rm{p}}}_{0}\cong \frac{4}{{{\rm{r}}}^{2}}(\frac{1}{2}\cdot \frac{1}{2\pi }{\int }_{0}^{2\pi }{\rm{v}}(2{\rm{r}},\theta ){\rm{d}}\theta )-\frac{1}{2\pi }{\int }_{0}^{2\pi }{\rm{v}}({\rm{r}},\theta ){\rm{d}}\theta )$$

where $\frac{1}{2\pi }{\int }_{0}^{2\pi }{\rm{v}}({\rm{r}},\theta ){\rm{d}}\theta$ and $\frac{1}{2\pi }{\int }_{0}^{2\pi }{\rm{v}}(2{\rm{r}},\theta ){\rm{d}}\theta$ represent the average potentials on the middle ring and outer ring, respectively.

Since the disc and outer ring were shorted, the quasi-bipolar configuration was not a true bipolar configuration^26^. Its Laplacian potential was estimated as

15 $${{\rm{\Delta }}{\rm{p}}}_{0}=\frac{({{\rm{v}}}_{{\rm{or}}}+{{\rm{v}}}_{{\rm{0}}})}{2}-{{\rm{v}}}_{{\rm{mr}}}$$

where v_or_ represents the potential of the outer rings, v_mr_ represents the potential of the middle rings, and v_0_ represents the potential of the central disc.

(c) Laplacian potential of tri-polar concentric ring electrode

Figure 7(c) shows the nine-point arrangement with p_0_ to p_8_, which was used as an approximation to a tri-polar concentric ring electrode. The Laplacian at point p_0_ was obtained using the Taylor series expansion and the finite difference approximation methods as:

16 $$(\frac{{\partial }^{2}{\rm{v}}}{\partial {{\rm{x}}}^{2}}+\frac{{\partial }^{2}{\rm{v}}}{\partial {{\rm{y}}}^{2}}){|}_{{\rm{P}}0}={{\rm{\Delta }}{\rm{p}}}_{0}\cong \frac{1}{12{{\rm{r}}}^{2}}\{{\rm{16}}\sum _{{\rm{i}}={\rm{1}}}^{{\rm{4}}}{{\rm{v}}}_{{\rm{i}}}-{{\rm{60v}}}_{{\rm{0}}}-\sum _{{\rm{i}}={\rm{5}}}^{{\rm{8}}}{{\rm{v}}}_{{\rm{i}}}\}$$

If we calculate the potential along the circle of radius 2r and r around the point p_0_ with the Taylor expansion, the approximation Laplacian to a tri-polar concentric ring electrode is as:

17 $${{\rm{\Delta }}{\rm{p}}}_{0}\cong \frac{1}{3{{\rm{r}}}^{2}}\{16(\frac{1}{2\pi }{\int }_{0}^{2\pi }{\rm{v}}({\rm{r}},\theta ){\rm{d}}\theta -{{\rm{v}}}_{0})-(\frac{1}{2\pi }{\int }_{0}^{2\pi }{\rm{v}}(2{\rm{r}},\theta ){\rm{d}}\theta -{{\rm{v}}}_{0})\}$$

where $\frac{1}{2\pi }{\int }_{0}^{2\pi }{\rm{v}}({\rm{r}},\theta ){\rm{d}}\theta$ and $\frac{1}{2\pi }{\int }_{0}^{2\pi }{\rm{v}}(2{\rm{r}},\theta ){\rm{d}}\theta$ represent the average potentials on the middle ring and outer ring, respectively.

#### Numerical simulation

COMSOL Multiphysics 5.0 (COMSOL Inc., Sweden), a finite analysis solver and simulation software, was used to calculate the surface potential detected by the each of the four types of electrodes.

#### Consistency of EHG signals between the four electrodes

The signal source of EHG on the dipole band model was calculated from formula (5), with the dipole band moving from the fundus to cervix of the uterus and returning to the fundus, as shown in Fig. 6(a). The EHG signals were then recorded by each of the four electrodes, with its amplitude and variation trend compared to evaluate the consistency of electrodes.

#### Attenuation of the potential with a moving dipole band

The local sensitivity^34^ of the four types of electrodes with different outer ring radius can be assessed by either moving the electrode or the dipole band. Because the estimation of the spatial resolution of the electrodes depends on the relative position between the electrode and dipole bands, positioning the electrode at different sites would lead to the same results with moving the dipole band in estimating the spatial resolution of the electrodes. In our study, the dipole band moved along the y-z plane (with a step size of 5 mm on the y axis from −30 to 30 mm and z axis from −30 to 30 mm), as shown Fig. 6(b). The position of the dipole band on x axis was kept constant while it was moving on the y-z plane along each path. Six noise dipoles were placed at random locations toward the positive direction of x axis. The distance between the origin and the location of dipole band at 20 dB attenuation was obtained, allowing the comparison of local sensitivity between electrodes. The shorter the distance, the more sensitive the electrode is.

#### Effects of fat and muscle thickness on EHG signal

After the optimal electrodes were determined based on the above assessment of the consistency and the local sensitivity ability, they were studied further to investigate the effects of fat and muscle thickness on EHG signals. Their Laplacian potentials were calculated and compared with the fat thickness from 0 to 30 mm and a fixed muscle thickness of 8 mm, and with the muscle thickness from 0 to 16 mm and a fixed fat thickness of 15 mm, respectively. In addition, signal attenuation values of the selected electrodes with different outer ring radius were estimated on the abdomen model with different fat and muscle thickness. The outer ring radius was 6, 10, 15, 20 and 25 mm, and the middle ring radius was 3, 5, 7.5, 10 and 12.5 mm.

### Data availability

All data generated or analyzed during the current study are available from the corresponding author on reasonable request.
